# CHANGES IN HEALTH CARE UTILIZATION FOR LOW-SES ADULTS NEAR RETIREMENT AFTER THE ACA MEDICAID EXPANSION

**DOI:** 10.1093/geroni/igz038.043

**Published:** 2019-11-08

**Authors:** Renuka Tipirneni, Kenneth M Langa, Ryan J McCammon, Kara Zivin, Jamie Luster, John Z Ayanian

**Affiliations:** University of Michigan Medical School, Ann Arbor, Michigan, United States

## Abstract

Low-SES Americans approaching retirement are experiencing rising morbidity and mortality. We examined longitudinal changes in health care access, utilization, and health for low-SES adults age 55-64 before (2010-2012) and after (2014-2016) ACA Medicaid expansion using the HRS. With a longitudinal difference-in-differences (DID) approach adjusting for demographics and the complex survey design, we found that low-SES adults age 55-64 had increased rates of Medicaid coverage (+10.7 percentage points [pp] in expansion states, +3.4 pp in non-expansion states, DID +7.3 pp) and increased likelihood of hospitalizations (+9.9 pp in expansion states, -1.2 pp in non-expansion states, DID +11.1 pp) in Medicaid expansion compared with non-expansion states. There were no other significant differences in access, utilization or health trends between expansion and non-expansion states. After Medicaid expansion, low-SES adults age 55-64 were more likely to be hospitalized, suggesting poorer baseline access to chronic disease management and associated pent-up demand for health care services.

